# Pulmonary Aspergilloma: A Treatment Challenge in Sub-Saharan Africa

**DOI:** 10.1371/journal.pntd.0002352

**Published:** 2013-10-24

**Authors:** Christian Pohl, Levan Jugheli, Fredrick Haraka, Elirehema Mfinanga, Khadija Said, Klaus Reither

**Affiliations:** 1 Ifakara Health Institute, Bagamoyo Research and Training Centre, Bagamoyo, United Republic of Tanzania; 2 Swiss Tropical and Public Health Institute, Basel, Switzerland; 3 University of Basel, Basel, Switzerland; University of California San Diego School of Medicine, United States of America

## Presentation of Case

**Figure 1:**
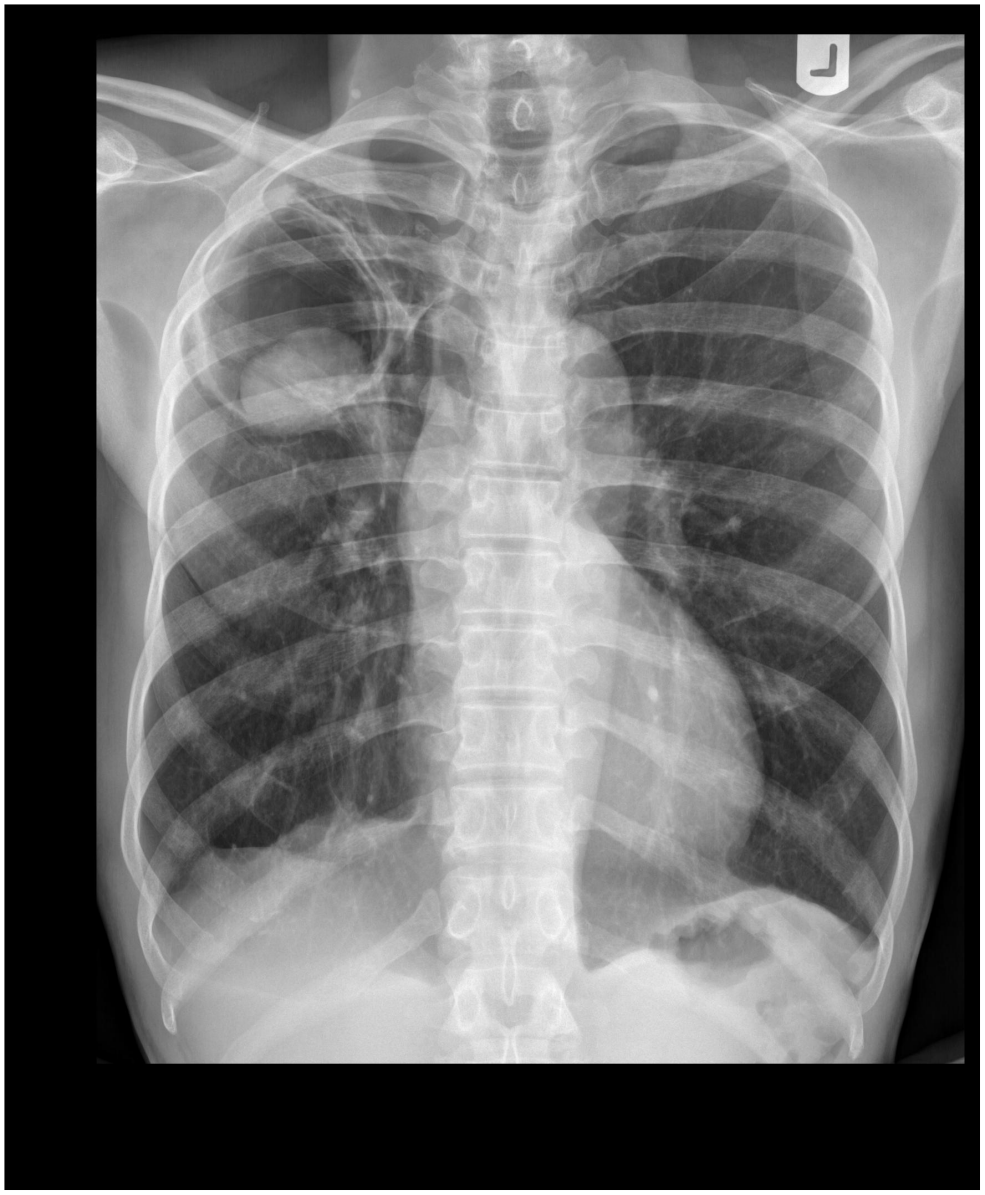
Chest radiography showing a fungus ball with an air crescent in the right upper lobe.

**Figure 2:**
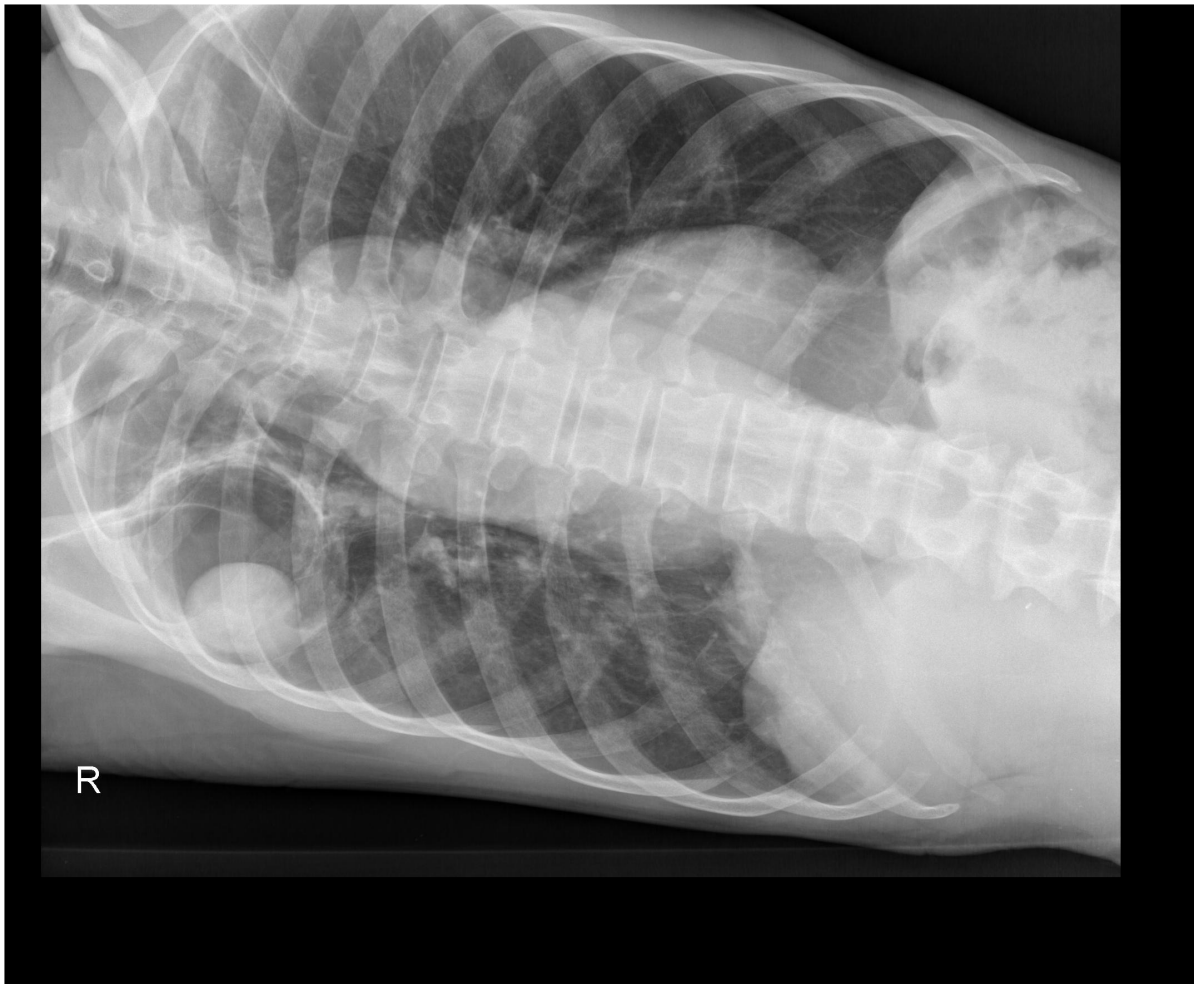
Chest radiography in supine position showing a change of position of the fungus ball.

In April 2011, a 68-year-old man presented himself at our research clinic in rural Tanzania with a three-month persistent productive cough, chest pain, night sweats, and recurrent nonmassive haemoptysis. He denied fever, night sweats, weight loss, and any recent contact with a known tuberculosis case. A prior treatment with a broad-spectrum antibiotic had not been successful. The patient reported having been treated for tuberculosis, diagnosed by sputum smear ten years ago. On examination he was afebrile, in a reduced general condition with a body mass index of 17.1 kg/m^2^. Other findings were bilateral reduced breath sounds and mild clubbing. Testing for HIV with two rapid tests (SD Bioline HIV 1/2 3.0 and Determine HIV-1/2) was negative. Posteroanterior chest radiography in an erect position showed a cavity of 60 mm×73 mm diameter in the right upper lobe containing an intracavitary focal mass of 47 mm×31 mm diameter with adjacent moon-shaped radiolucency (Figure 1). A second radiography in a supine position showed a changed position of this focal mass (Figure 2). A chest x-ray of the previous TB episode was not available for comparison. Smear microscopy of early morning and spot sputum after Ziehl-Neelsen stain was negative for acid-fast bacilli. A nucleic acid amplification test (Xpert MTB/RIF) did not detect *Mycobacterium tuberculosis*. Both the early morning and spot sputum samples were subsequently cultured on solid (Löwenstein Jensen) and in liquid (MGIT) media, neither of which showed mycobacterial growth after eight and six weeks, respectively. In order to isolate *Aspergillus*, a sabouraud dextrose agar was inoculated directly with sputum, but only *Enterobacter cloacae* could be found. Due to lack of facilities, neither an ELISA for IgG antibodies to *Aspergillus* nor an Aspergillus precipitin test could be performed.

Based on radiographies, a diagnosis of single pulmonary aspergilloma was established, but we could not exclude that the symptoms were caused by tuberculosis reactivation or reinfection. With negative sputum smears, chest radiography findings consistent with tuberculosis, and a lack of response to a trial broad-spectrum antimicrobial agent, our patient fulfilled the WHO criteria for sputum smear–negative tuberculosis. Without options for surgical treatment of pulmonary aspergilloma, we were now faced with the decision of either starting medication for pulmonary aspergilloma or sending the patient for tuberculosis treatment. Considering unavailability of sputum culture results at this point and our setting with high prevalence of tuberculosis, we presented the case to the National Tuberculosis and Leprosy Program (NTLP) which initiated six-month standard tuberculosis treatment according to national guidelines, which implies prescription of rifampin, isoniazid, ethambutol, and pyrazinamide for two months, followed by rifampin and isoniazid for four months. After six months of antituberculosis therapy, the patient was still in a reduced condition, complaining about productive cough and chest pain, but no haemoptysis or night sweats. His body mass index had increased to 19.2 kg/m^2^. Radiographies, however, did not show any improvement. Because of the persistent symptomology, the patient was subsequently started on antifungal treatment with itraconazole 200 mg daily for six months. At the end of this period, the treatment was extended for another six months because the patient had reported that the dispensary had not been able to provide him with medication continuously and therefore he had not been able to take medication for the last three months of treatment. At the last follow-up in October 2012 we noticed a clinical improvement of the chest pain and no productive cough despite a follow-up radiography not showing any changes.

## Case Discussion

### Pulmonary Aspergilloma

Since the common term *mycetoma* applies accurately to soft tissue infections, it is more precise to use *pulmonary aspergilloma* to describe an intracavitary fungal mycelial growth in the lung. Pulmonary aspergilloma caused by *A. fumigatus* is the most widespread of the noninvasive forms of pulmonary aspergillosis and develops in preexisting lung cavities, most frequently tuberculous caverns as seen in our case [1], [2]. The aspergilloma (fungus ball) consists of masses of fungal mycelia, inflammatory cells, fibrin, mucus, and tissue debris [3]. The diagnosis is usually made clinically and radiographically without lung biopsy. Mild haemoptysis is commonly reported as the main symptom, but most patients are asymptomatic. Bleeding is usually caused by local invasion and endotoxic or mechanical irritation of exposed bronchial blood vessels. Symptoms like cough and dyspnoea are more likely related to underlying diseases, making a clear diagnosis difficult [1]. As with our patient, 50% of pulmonary aspergilloma sputum cultures for *Aspergillus* spp. are negative [2]. Serum IgG antibodies to *Aspergillus* are positive in most cases, but may be false negative in patients under corticosteroid therapy or in rare cases of pulmonary aspergilloma caused by other species than *A. fumigatus* [1], [3]. Chest radiographies show intracavitary mass (fungus ball) with an air crescent (Monod sign) in about two-thirds of the cases [2], [4]. This pattern is mostly localized in the upper lobe and can change position with gravity like in our radiographies. Computed tomography can be useful to detect smaller lesions that may not be apparent on conventional radiographies. An increase of wall thickness of the preexisting cavity suggests secondary infection [4]. Risk factors for poor prognosis of pulmonary aspergilloma include the severity of the underlying lung disease, increase in size or number of lesions, and immunosuppression [1], [3].

### Treatment Difficulties

The first major decision is whether therapy of the aspergilloma is required. With 10% of asymptomatic cases resolving spontaneously, treatment usually becomes necessary only when the patient develops symptoms [5]. Definitive treatment for an aspergilloma is surgery of the affected lung, which is indicated in case of recurrent or severe haemoptysis. However, surgical resection of the affected lung is associated with considerable morbidity and mortality. The mortality rates for surgical resection in symptomatic patients vary between 4–8%, the morbidity approaches 25% [6]–[8]. These rates can be explained partly because of the poor pulmonary function of many patients with aspergilloma. Additionally, the disease is rarely confined to the cavitary lesion seen on chest radiography and the adjacent lung and pleura are also involved in the inflammatory response [7]. Lobectomy, segmentectomy, pneumonectomy, and cavernostomy can be used as surgical procedures. The risks of compromised pulmonary function, bronchopleural fistula, bleeding, and infection of the pleural space may outweigh the benefits, depending on the individual patient. In inoperable patients, primary treatment with antifungal agents seems to be an option. To date, there is no consistent evidence about the appropriate pharmacological treatment of single pulmonary aspergilloma. The data guiding the management of the disease are based on uncontrolled trials and case reports [9]. Inhaled, intracavitary, and endobronchial instillations of antifungal agents have shown inconsistent success [1], [10]. Among oral antifungals, itraconazole seems to be most effective due to its high tissue penetration, but only small studies have shown benefit so far. Because of its slow effect, treatment regimens have to last at least six months. The rates of symptomatic improvement vary from 61% to 71%, and those of radiological improvement from 14% to 23% [9]–[12]. However, due to the unpredictable natural history of aspergilloma, it is difficult to attribute clinical improvement to a specific treatment. Parallel treatment with standard tuberculosis medication should be avoided, because of the well-documented interaction between itraconazole and rifampin which may lead to therapeutic failure [13]. The role of newer antifungal azoles such as voriconazole in the treatment of aspergilloma has yet to be determined [1].

## The Presenting Case

There is no epidemiological data on pulmonary aspergilloma in Tanzania and sub-Saharan Africa. However, the high burden of tuberculosis in these countries suggests a considerable number of unreported cases. The case described here should not illustrate a new treatment approach but emphasize the limitations and challenges in diagnosis and treatment due to resource limitations, the tuberculosis epidemic, and lack of consensus on therapeutic approach. The means used in this case surpassed the resources of an average rural setting, where we would have encountered even more limitations. Our initial diagnosis was purely based on the typical radiological findings illustrated in Figure 1 and Figure 2. Recurrent nonmassive haemoptysis and other clinical symptoms at the first consultation were consistent with pulmonary aspergilloma and other pulmonary conditions, including tuberculosis. Unfortunately, we could not compare radiographies with the tuberculosis episode that the patient had ten years before and distinguish between new and old radiological manifestations. Consequently, our case fulfilled all criteria for a sputum smear–negative case according to national standards [14] and the international standards for tuberculosis care in high epidemic countries [15], and the NTLP decided to start treatment despite of the unavailability of mycobacterial cultures at this point. If we had attributed the symptoms purely to pulmonary aspergilloma, surgical resection would have been the best treatment choice. Missing options of surgical treatment due to lack of resources and epidemiological concerns led to the decision to treat pharmacologically, first for tuberculosis and subsequently for pulmonary aspergilloma. With total treatment duration of at least 12 months, it was difficult to guarantee proper adherence to both treatments. In our case, insufficient drug supply, as well as logistical difficulties in making the antifungal drugs available for the patient, led us to extend the antifungal therapy for another six months. It remains unclear if the treatment with tuberculostatica, antifungal medication, or the natural history of pulmonary aspergilloma caused the clinical improvement of the patient, whose symptoms disappeared except residual chest pain. Retrospectively, although it was initially not possible to exclude a smear-negative tuberculosis case, treatment of pulmonary aspergilloma should have been given priority while TB treatment should have been started only in the case of positive cultures.

The challenges in differential diagnosis and therapy of pulmonary aspergilloma demonstrated in this case underline the necessity for incorporating management of this frequently neglected TB-associated disease into national TB treatment guidelines in endemic countries.

**Learning Points**

- Pulmonary aspergilloma develops most frequently in residual tuberculous cavities.
- Symptoms are often unspecific, making imaging the cornerstone of the diagnosis.
- A mobile intracavitary mass with an air crescent are pathognomonic x-ray findings.
- In symptomatic patients, surgical resection is the treatment of choice.
- Antifungal treatment can be considered as a therapeutic option, if surgery is not possible.
- Parallel treatment with rifampin and itraconazole should be avoided because of drug interactions.
